# Biventricular Levitronix CentriMag Assist Device: A “Bridge to Recovery” Solution in Patients with Acute Fulminant Myocarditis

**DOI:** 10.1155/2012/907490

**Published:** 2012-11-27

**Authors:** Marcel Vollroth, Markus J. Barten, Friedrich W. Mohr, Jens Garbade

**Affiliations:** Department of Cardiac Surgery, Heartcenter Leipzig University, Struempellstraße 39, 04289 Leipzig, Germany

## Abstract

Fulminant myocarditis (FM) represents a crucial cardiac pathology with extensive hemodynamic compromise occurring in a previously healthy patient. Early death occurs because of acute cardiac decompensation from inflammation, necrosis, and myocytolysis. Nevertheless, in this situation implantation of an extracorporeal circulatory support system may ensure cardiac recovery. We herein report our experience using a biventricular Levitronix CentriMag system for bridge to recovery.

## 1. Introduction

Fulminant myocarditis (FM) represents a crucial cardiac pathology characterized by rapid and extensive hemodynamic compromise occurring in a previously healthy patient [1]. In cases of nonfulminate myocarditis, conventional heart failure management strategies can stabilize the patient until complete recovery. For more fulminant course early death occurs because of acute cardiac decompensation from inflammation, necrosis, and myocytolysis. Nevertheless, in this situation implantation of an extracorporeal circulatory support system may ensure cardiac recovery [2]. In most cases of FM, ventricular recovery can be expected within several weeks of onset. Time of implantation, device selection, and perioperative strategy must be considered before multiorgan failure occurs [3]. We herein report our first experiences using a nonpulsatile biventricular Levitronix CentriMag system for bridge to recovery.

## 2. Case report

A 49-year-old previously healthy woman presented to a local emergency department because of recurrent fatigue, dyspnoea, and chest pain within the last three weeks. She described a “flu-like” upper respiratory infection for the past month that progressively worsened to these specific symptoms. The echocardiographic evaluation showed ejection fraction (EF) about 10% with biventricular failure. Despite pathologic ECG and highly elevated cardiac troponin, urgent cardiac catheterization detected only mild coronary sclerosis. During this procedure she progressed to circulatory collapse with the need for highly inotropic support and mechanical ventilation. For higher level of care the lady was transferred to our institution. 

Due to rapid aggravation of her condition while admission at our ICU, we initiated intraaortic balloon pump (IABP) implantation via the right femoral artery and venoarterial Extracorporeal Membrane Oxygenation (ECMO) implantation through the right femoral vein and right axillary artery. Despite maximum hemodynamic support, the heart showed no evidence of recovery. Furthermore the lady progressed to multiple organ failure including highly elevated lactate level, acute renal failure, and hepatic insufficiency. Our Heart Team decided on urgent biventricular CentriMag (Levitronix, Waltham, MA) implantation to decompress and support both ventricles. 

**Figure 1:**
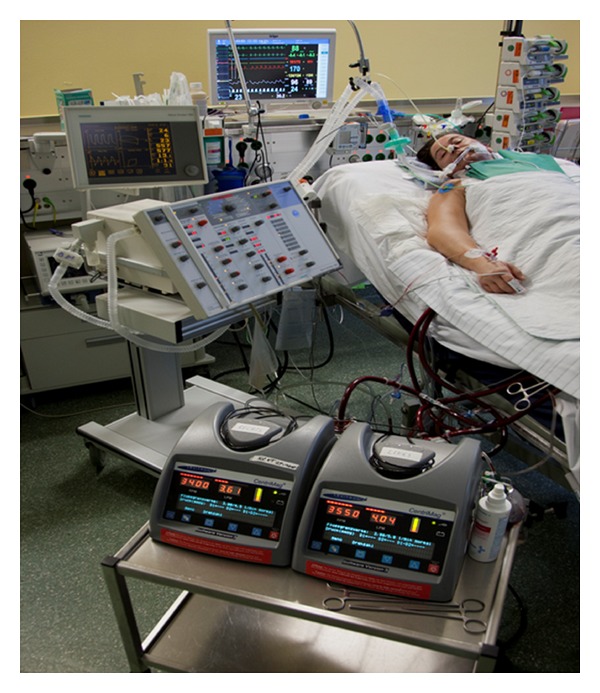
Patient on postoperative day 1. In front the two CentriMag pumps for biventricular unloading.

Through a median sternotomy the right atrium and aorta were cannulated and the patient was transferred from ECMO to cardiopulmonary bypass (CPB). A left ventricular 23 F cannula and a 22 F right atrial p-VAD cannula were placed for biventricular outflow into the CentriMag pumps. The left apex cannulation was chosen for maximum flow and complete left ventricular decompression. For return flow to pulmonary and systemic circulation the pulmonary artery and the ascending aorta were anastomosed with two 18 F sealed inflow cannulas. The patient was then weaned from CPB to BVAD support. The ECMO cannulas were removed and the femoral vessels repaired. With moderate inotropic support the patient was administrated to our ICU (Figure 1). Intravenous continuous heparine was introduced to achieve an activating clotting time of 140–160 s.

The postinterventional course was complicated by renal failure and neurologic morbidity necessitating continuous dialysis and tracheotomy. Left and right ventricular functions were daily assessed by transesophageal echocardiogram (TEE), while contractility and aortic valve opening were studied. Recovery of left ventricular contractility began on postimplantation day seven. We considered BVAD explantation when full recovery of biventricular contractility and aortic valve opening were observed. Weaning started on hospital on day 21, with the patient's condition and cardiac function improving to an ejection fraction (EF) of 50% in sinus rhythm. The CentriMag BVAD was removed nine days later without the need for additional CPB. Transesophageal control echocardiogram showed an EF >60% and sufficient right ventricular function. Ten days later she was discharged to rehabilitation in a stable condition.

The patient had signs of FM on histological findings with foci myocyte necrosis and lymphocytic infiltration with interstitial oedema. In spite of serologic studies and viral genome research on myocardial biopsies, including in situ hybridization and polymerase chain reaction, pathogenic agents were not identified.

## 3. Discussion 

FM is clinically characterized by distinct onset of cardiac symptoms in otherwise healthy patients after nonspecific flu-like symptoms rapidly resulting in severe ventricular dysfunction and cardiogenic shock. Most of these patients with acute fulminant myocarditis are stabilized with heart failure management (inotropic support, afterload reduction, and IABP support) and recover within a few weeks [4]. However, in some serious cases patients necessitate mechanical support. Recently, some colleagues postulated that ECMO should be preferred due to short time recovery. ECMO support offers many advantages (easy and rapid insertion), but sufficient biventricular unloading remains challenging. 

For our case percutaneous cannulation and emergency implementation of ECMO was initially the therapy of choice due to her life-threatening condition, maybe as a bridge to decision. With cardiac nonrecovery evident and progressive endorgan dysfunction, support was escalated to biventricular nonpulsatile CentriMag assist device implantation. 

Arguments in favour of BVAD shortterm support are better unloading of both ventricles, a primary condition for recovery from fulminant myocarditis, and avoidance of significant morbidities associated with prolonged ECMO support [5]. 

## 4. Conclusion

Successful treatment of fulminant myocarditis with severe hemodynamic compromise and end organ failure is possible with early CentriMag biventricular assist device implantation. In most cases it facilitates completely cardiac recovery.
